# Incidence and Severity of Coronary Artery Disease in Patients with Atrial Fibrillation Undergoing First-Time Coronary Angiography

**DOI:** 10.1371/journal.pone.0024964

**Published:** 2011-09-21

**Authors:** Stefan Kralev, Kathrin Schneider, Siegfried Lang, Tim Süselbeck, Martin Borggrefe

**Affiliations:** Department of Medicine, Faculty of Medicine Mannheim, University of Heidelberg, Mannheim, Germany; University of Modena and Reggio Emilia, Italy

## Abstract

**Background:**

In standard reference sources, the incidence of coronary artery disease (CAD) in patients with atrial fibrillation (AF) ranged between 24 and 46.5%. Since then, the incidence of cardiovascular risk factors (CRF) has increased and modern treatment strategies (“pill in the pocket”) are only applicable to patients without structural heart disease. The aim of this study was to investigate the incidence and severity of CAD in patients with AF.

**Methods:**

From January 2005 until December 2009, we included 261 consecutive patients admitted to hospital with paroxysmal, persistent or permanent AF in this prospective study. All patients underwent coronary angiography and the Framingham risk score (FRS) was calculated. Patients with previously diagnosed or previously excluded CAD were excluded.

**Results:**

The overall incidence of CAD in patients presenting with AF was 34%; in patients >70 years, the incidence of CAD was 41%. The incidence of patients undergoing a percutaneous coronary intervention (PCI) or coronary artery bypass graft (CABG) was 21%. Patients with CAD were older (73±8 years *vs* 68±10 years, p = 0.001), had significantly more frequent hypercholesterolemia (60% *vs* 30%, p<0.001), were more frequent smokers (26% *vs* 13%, p = 0.017) and suffered from angina more often (37% *vs* 2%, p<0.001). There was a significant linear trend among the FRS categories in percentage and the prevalence of CAD and PCI/CABG (p<0.0001).

**Conclusions:**

The overall incidence of CAD in patients presenting with AF was relatively high at 34%; the incidence of PCI/CABG was 21%. Based upon increasing CRF in the western world, we recommend a careful investigation respecting the FRS to either definitely exclude or establish an early diagnosis of CAD – which could contribute to an early and safe therapeutic strategy considering type Ic antiarrhythmics and oral anticoagulation.

## Introduction

Atrial fibrillation (AF) is the most common cardiac arrhythmia, affecting 2.3 million individuals in the USA and 4.5 million people in the EU [1]. AF is associated with multiple symptoms, significant mortality and morbidity and decreased quality of life [2], [3].

Importantly, after acute myocardial infarction, development of AF is associated with a worse prognosis [4]. Common cardiovascular risk factors also give support to associate coronary artery disease (CAD) and AF. Previous studies have found that hypertension, diabetes and obesity are conditions predisposing to AF [5], although in the case of diabetes conflicting data have been reported [6]. The Framingham study suggested that angina predisposed to AF and that the association of AF with CAD was stronger in men [7]. Despite the high prevalence of CAD in patients with AF of 18–46.5% [8]–[12], the prevalence of AF among patients with proven CAD is extremely low, at 0.2–5% [13]–[16]. By contrast, a survey of historic literature by Zipes indicates that AF commonly occurs in patients with CAD [17].

Over recent years, the incidence of cardiovascular risk factors of the western world have dramatically increased and this trend is projected to continue. Newer studies report different incidences of CAD in patients with AF but also focus on different patient groups [12], [18], so data is lacking on the overall incidence of CAD in AF patients in the modern era of cardiology. Further, the possibility of specific antiarrhythmic therapy has increased the interest in investigating patients with AF for CAD [19]. According to the American Heart Association/American College of Cardiology (AHA/ACC) guidelines, there is a class I recommendation for flecainide and propafenone for pharmacological cardioversion and a class IIa recommendation for flecainide and propafenone for selected patients (“pill-in-the-pocket”) without structural heart disease [20].

The aim of the present study was to investigate invasively the incidence and severity of CAD in patients with AF, presenting without previously diagnosed or previously excluded CAD.

## Methods

The study complies with the Declaration of Helsinki and all patients gave written informed consent. This study was approved by the Medical Ethic Commission II, Faculty of Medicine Mannheim, University of Heidelberg. The study was also performed in accordance with federal laws and regulations, international accreditation standards and institutional policies.

### Patient selection

From January 2005 until December 2009 we included 261 consecutive patients admitted to hospital with paroxysmal, persistent or permanent AF in this prospective study. Independent of the rhythm on admission, patients with previously known AF as well as patients with diagnosed AF on admission were included. Patients with previously diagnosed or previously excluded CAD, acute coronary syndromes and dilated or hypertrophic cardiomyopathy were excluded.

All patients underwent coronary angiography and the Framingham risk score (FRS) was calculated. Type of AF (paroxysmal, permanent or persistent), baseline clinical data, cardiac risk factors, left ventricular (LV) systolic function, existence of angina pectoris, existence of hyperthyreosis, above-average alcohol consumption and medication at discharge were (among others) recorded. In patients diagnosed with CAD, the number of diseased vessels and the affected coronary artery [left anterior descending (LAD), left circumflex artery (LCX) and right coronary artery (RCA)] were assessed. Diagnosis of CAD and indication for percutaneous coronary intervention (PCI) was performed according to the current AHA/ACC guidelines [21]. In patients undergoing PCI the target vessel was recorded. Additionally, patients undergoing coronary artery bypass graft (CABG) were noted.

### Statistical analysis

For normally distributed data the unpaired Student's *t* test was applied. The nonparametric Mann-Whitney U test was used when the data deviated from a Gaussian distribution as tested by the Kolmogorov-Smirnov test. Comparisons of categorial variables were carried out by χ^2^ and Fisher's exact tests. Data are presented as mean ± standard deviation (SD) or number and percentage for categorical variables. Values of p<0.05 (two-tailed) were considered statistically significant. The calculations were performed using SPSS-Software (SPSS-Software GmbH, München, Germany) and InStat (GraphPad Software, San Diego, USA).

## Results

**Figure 1:**
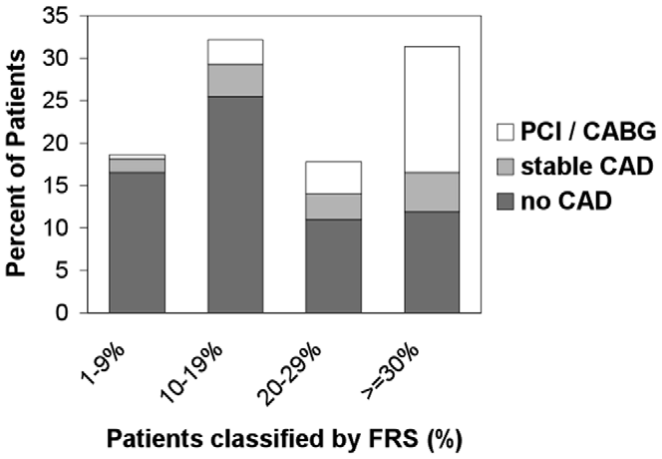
Significant linear trend among the FRS categories in % and the prevalence of CAD and PCI/CABG (p<0.0001). CABG = Coronary Artery Bypass Graft, CAD = Coronary Artery Disease, FRS = Framingham Risk Score, PCI = Percutaneous Coronary Intervention.

**Figure 2:**
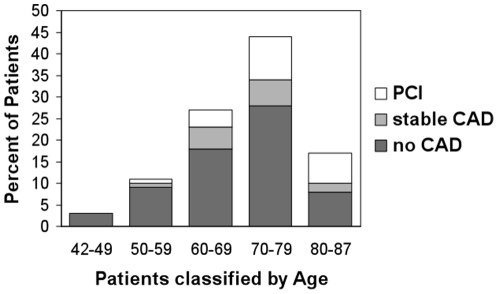
Incidence and severity of coronary artery disease in patients presenting with atrial fibrillation according to age. CAD = Coronary Artery Disease, PCI = Percutaneous Coronary Intervention.

**Figure 3:**
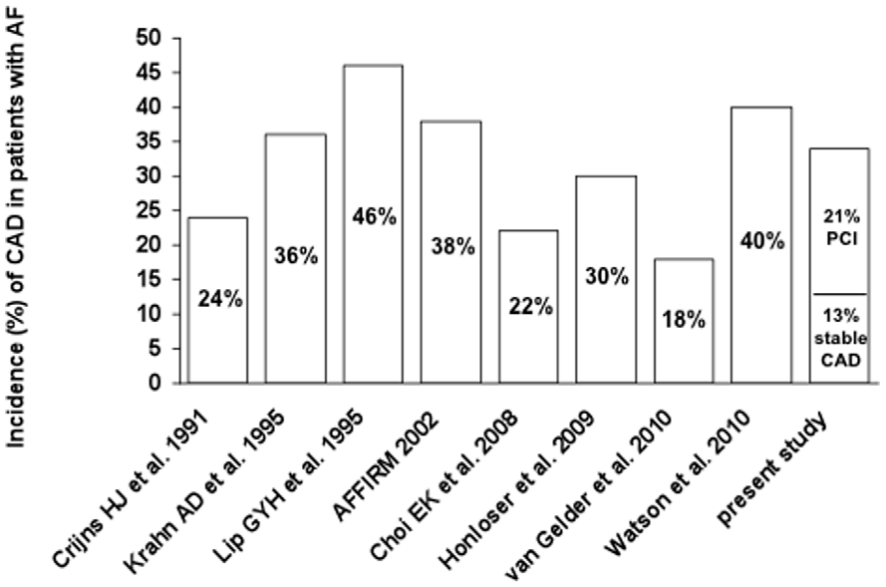
Overview of reported incidences of coronary artery disease in patients presenting with atrial fibrillation. AF = Atrial Fibrillation, CAD = Coronary Artery Disease, PCI = Percutaneous Coronary Intervention.

Overall, 261 patients with AF and unknown history of CAD were analysed. In 171 patients (65.5%) CAD was excluded. A stable CAD was diagnosed in 34 patients (13%) and PCI or CABG was performed in 56 patients (21%). No patient died, developed a stroke or a myocardial infarction. Nine patients underwent inhospital CABG. Among the patients with newly detected CAD, the percentage of patients undergoing PCI/CABG was 62.2%. Patients with CAD were older (73±8 years *vs* 68±10 years, p = 0.001), had significantly more frequent hypercholesterolemia (60% *vs* 30%, p<0.001), were more frequent smokers (26% *vs* 13%, p = 0.017) and suffered from angina more often (37% *vs* 2%, p<0.001). There was a significant linear trend among the FRS categories in % and the prevalence of CAD and PCI/CABG (p<0.0001) with more cases of PCI and CAD at elevated FRS levels. The association between the FRS and % of CAD in AF is presented in Figure 1. 61 patients (23%) in this study were >70 years old, and of these 25 (41%) presented with a CAD (Figure 2 shows the age-dependent distribution). The incidence of male gender was higher among patients with detected CAD, but did not reach statistical significance (72% *vs* 60%, p = 0.057). Therapy with a class Ic antiarrhythmic drug was initiated in 39 of 171 patients (23%). The comparison of patients with and without CAD is shown in Table 1; the overall incidence of CAD (in comparison to previous studies) is depicted in Figure 3.

**Table 1:** Comparison of patients without CAD and patients with CAD.

Patient characteristic	Without CAD(n = 171)	With CAD(n = 90)	P-Value
Age ± SD (Years)	68±10	73±8	**0.001**
Male sex	102 [60%]	65 [72%]	0.057
***Type of AF***			
Paroxysmal	77 [45%]	47 [52%]	0.30
Persistent	47 [27%]	16 [18%]	0.095
Permanent	47 [27%]	27 [30%]	0.67
***Risk factors***			
Smoking	23 [13%]	23 [26%]	**0.017**
Hypercholesterolemia	52 [30%]	54 [60%]	**<0.001**
Hypertension	113 [66%]	65 [72%]	0.33
Obesity	38 [22%]	20 [22%]	1.0
Familiar history of CAD	16 [9%]	9 [10%]	0.83
Diabetes mellitus	25 [15%]	21 [23%]	0.11
***Left ventricular systolic function*** *			
EF≥55%	119 [70%]	52 [58%]	0.075
EF 45–54%	34 [20%]	22 [24%]	0.43
EF 30–44%	12 [7%]	11 [12%]	0.17
EF<30%	6 [4%]	5 [6%]	0.52
***Other data***			
Angina pectoris	3 [2%]	33 [37%]	**<0.001**
Above-average alcohol consumption	5 [3%]	2 [2%]	1.0
Hyperthyreosis	14 [8%]	14 [16%]	0.091
***Medication at discharge***			
Aspirin	24 [14%]	47 [52%]	**<0.001**
Clopidogrel	3 [2%]	39 [43%]	**<0.001**
Phenprocoumon	119 [70%]	32 [36%]	**<0.001**
Beta-blockers	118 [69%]	62 [69%]	1.0
Calcium channel antagonists	26 [15%]	17 [19%]	0.48
Digitalis glycosides	71 [42%]	44 [49%]	0.29
Amiodarone	7 [4%]	2 [2%]	0.72
Flecainide	23 [13%]	5 [6%]	0.059
Propafenone	16 [9%]	1 [1%]	**0.008**

AF = Atrial Fibrillation; CAD = Coronary Artery Disease; EF = Ejection Fraction; SD = Standard Deviation;

*Significant “within-group” trend towards EF≥55%.

Comparing patients with stable CAD and patients undergoing PCI/CABG (Table 2), patients with stable CAD presented more often with one-vessel disease (79% *vs* 34%, p<0.0001) and significantly less frequently with two- and three-vessel disease. In patients who underwent PCI, CAD was more frequently detected in the RCA (62% *vs* 26%, p = 0.001). Regarding age, gender and single cardiac risk factors, no differences were found between patients with stable CAD and patients undergoing PCI/CABG.

**Table 2:** Comparison of patients with stable CAD and patients undergoing PCI/CABG.

Patient characteristics	Stable CAD(n = 34)	PCI/CABG(n = 56)	P-Value
Age ± SD (Years)	72±8	74±8	0.24
Male sex	22 [65%]	43 [77%]	0.23
***Risk factors***			
Smoking	9 [26%]	14 [25%]	1.0
Hypercholesterolemia	17 [50%]	37 [66%]	0.18
Hypertension	22 [65%]	43 [77%]	0.23
Obesity	9 [26%]	11 [20%]	0.6
Familiar history of CAD	4 [12%]	5 [9%]	0.73
Diabetes mellitus	5 [15%]	16 [29%]	0.15
Angina pectoris	10 [29%]	23 [41%]	0.37
***Left ventricular systolic function*** *			
EF≥55%	19 [56%]	33 [59%]	0.83
EF 45–54%	8 [24%]	17 [30%]	0.63
EF 30–44%	6 [18%]	2 [4%]	0.058
EF<30%	1 [3%]	4 [7%]	0.65
***Number of vessel disease***			
1-vessel disease	27 [79%]	19 [34%]	**<0.0001**
2-vessel disease	4 [12%]	20 [36%]	**0.015**
3-vessel disease	3 [9%]	17 [30%]	**0.019**
***Localisation of CAD*** †			
LAD	23 [68%]	45 [80%]	0.21
LCX	12 [35%]	30 [54%]	0.13
RCA	9 [26%]	35 [62%]	**0.001**
***Target vessel*** ‡			
LAD		27 [48%]	
LCX		8 [14%]	
RCA		12 [21%]	
***CABG***		9 [16%]	

AF = Atrial Fibrillation; CAD = Coronary Artery Disease; CABG = Coronary Artery Bypass Graft; EF = Ejection Fraction; LAD = Left Anterior Descending Artery; LCX = Left Circumflex Artery; PCI = Percutaneous Coronary Intervention; RCA = Right Coronary Artery; SD = Standard Deviation;

*Significant “within-group” trend towards EF≥55%;

† No statistical significance was found regarding the “within-group” allocation of LAD, LCX and RCA;

‡ “Within-group” incidence of LAD significantly increased (P = 0.042).

**Figure 4:**
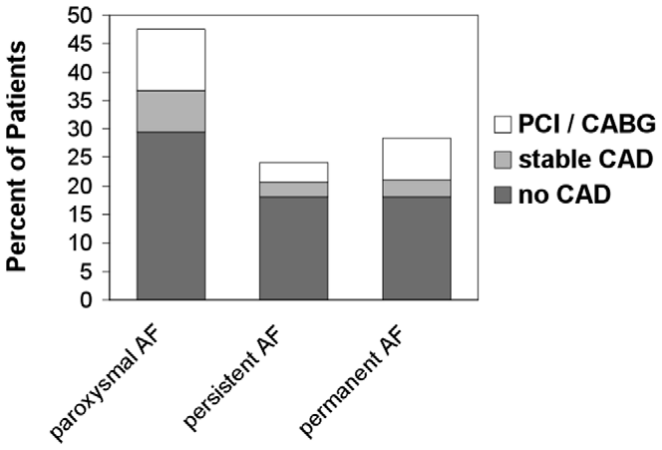
Prevalence and management of coronary artery disease (drug-treated stable CAD vs. PCI/CABG) according to subtype of atrial fibrillation did not differ significantly. AF = Atrial Fibrillation, CABG = Coronary Artery Bypass Graft, CAD = Coronary Artery Disease, PCI = Percutaneous Coronary Intervention.

**Figure 5:**
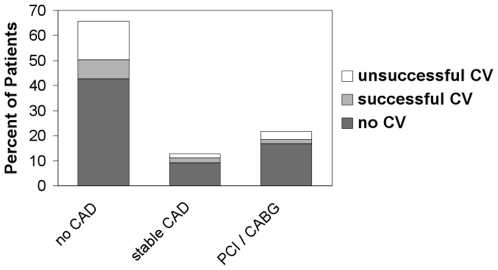
Unsuccessful but also successful electrical cardioversion were more frequently performed in patients without CAD than in patients with stable CAD or with PCI/CABG (p<0.01). CAD = Coronary Artery Disease, CABG = Coronary Artery Bypass Graft, CV = electrical Cardioversion, PCI = Percutaneous Coronary Intervention.

Patients without CAD and patients with stable CAD were pooled in one group and were compared with patients who underwent PCI/CABG (Table 3). Patients with PCI were older (74±8 years *vs* 69±10 years, p<0.001), were more frequently male (77% *vs* 60%, p = 0.028), had hypercholesterolemia more often (66% *vs* 34%, p<0.001) and had a significantly increased incidence of diabetes mellitus (29% *vs* 15%, p = 0.028). Patients with PCI also suffered from angina more often (41% *vs* 6%, p<0.001). The prevalence and management of CAD (drug-treated stable CAD vs. PCI/CABG) according to subtype of atrial fibrillation did not differ significantly (p = 0.79, χ^2^ test) and is depicted in Figure 4. No significant differences were found when the different patient groups (without CAD, stable CAD, PCI) were compared for LV systolic function, but in all groups there was a significant “within-group” trend towards ejection fraction (EF) ≥55% (Tables 1, 2, and 3). Unsuccessful but also successful electrical cardioversion were more frequently performed in patients without CAD than in patients with stable CAD or with PCI/CABG (p<0.01, Figure 5).

**Table 3:** Comparison of patients without/stable CAD and patients with PCI or CABG.

Patient characteristics	Without/stable CAD(n = 205)	PCI/CABG(n = 56)	P-Value
Age ± SD (Years)	69±10	74±8	**0.001**
Male sex	124 [60%]	43 [77%]	**0.028**
***Type of AF***			
Paroxysmal	96 [47%]	28 [50%]	0.76
Persistent	54 [26%]	9 [16%]	0.16
Permanent	55 [27%]	19 [34%]	0.32
***Risk factors***			
Smoking	32 [16%]	14 [25%]	0.12
Hypercholesterolemia	69 [34%]	37 [66%]	**<0.001**
Hypertension	135 [66%]	43 [77%]	0.15
Obesity	47 [23%]	11 [20%]	0.72
Familiar history of CAD	20 [10%]	5 [9%]	1.0
Diabetes mellitus	30 [15%]	16 [29%]	**0.028**
***Left ventricular systolic function*** *			
EF≥55%	138 [67%]	33 [59%]	0.27
EF 45–54%	42 [20%]	17 [30%]	0.15
EF 30–44%	18 [9%]	2 [4%]	0.26
EF<30%	7 [3%]	4 [7%]	0.26
***Other data***			
Angina pectoris	13 [6%]	23 [41%]	**<0.001**
Above-average alcohol consumption	6 [3%]	1 [2%]	1.0
Hyperthyreosis	21 [10%]	7 [12%]	0.63
***Medication at discharge***			
Aspirin	36 [18%]	35 [62%]	**<0.001**
Clopidogrel	8 [4%]	34 [61%]	**<0.001**
Phenprocoumon	135 [66%]	16 [29%]	**<0.001**
Beta-Blockers	136 [66%]	44 [79%]	0.10
Calcium channel antagonists	34 [17%]	9 [16%]	1.0
Digitalis glycosides	87 [42%]	28 [50%]	0.36
Amiodarone	9 [4%]	0	0.21
Flecainide	26 [13%]	2 [4%]	0.053
Propafenone	17 [8%]	0	0.028

AF = Atrial Fibrillation; CAD = Coronary Artery Disease; CABG = Coronary Artery Bypass Graft; EF = Ejection Fraction; SD = Standard Deviation;

*Significant “within-group” trend towards EF≥55%.

## Discussion

This single centre study reports on patients with AF undergoing first time coronary angiography, presenting data relating to the incidence and severity of CAD.

### AF and cardiovascular risk factors

The Framingham Heart Study [7] concluded that hypertension and diabetes are significantly associated with risk for AF. In addition, two other studies reported that hypertension, diabetes and obesity were conditions predisposing to AF [5]. Nevertheless, in the case of diabetes, data are contrasting. Whereas Movahed *et al.* [22] reported that diabetes is a strong independent risk factor for AF, Wilhelmsen *et al.* found no correlation between diabetes mellitus and AF [6]. In our study, only the comparison of patients without CAD or stable CAD *versus* patients undergoing PCI yielded a significantly higher incidence of diabetes in the PCI group (Table 3). Regarding other cardiovascular risk factors, patients with CAD (compared to patients without CAD) had significantly more frequent hyperlipidaemia, were more frequent smokers and were older. Importantly, the CHADS_2_ score (Congestive heart failure, Hypertension, Age >75, Diabetes, prior Stroke -doubled [23], [24]) or the refined CHA_2_DS_2_-VASc score (difference to CHADS_2_: AGE ≥75 years 2 points, AGE 65–74 years 1 point, Vascular disease [prior MI, peripheral artery disease or aortic plaque], Sex category) should be applied to enable a stratification of stroke risk [25]. In conclusion, at least a stress test is reasonable in AF patients with cardiovascular risk factors and, citing the guidelines, the presence of AF alone, without other stigmata of CAD, should prompt the cardiologist to search for causes of AF other than CAD ([20] page 11).

### AF and LV function

Since patients with severe CAD are very likely to have impaired LV systolic function, heart failure seems more likely to be a cause of AF. Most of the patients included in this study had an EF≥55%. There was a significant “within-group” trend (patient groups: without CAD, stable CAD, PCI) towards EF≥55% and no significant difference was found comparing the different patient groups with each other regarding the incidence of EF<30%, EF 30–44% or EF 45–54% (Tables 1, 2 and 3). In any event, in patients presenting with persistent signs of impaired LV dysfunction, coronary angiography and careful consideration about the underlying heart disease is recommended ([20] page 11). Ischemic cardiomyopathy, heart failure and neurohumoral activation are hypothesized to play an important mediating role in the development of new AF [5], [13] and previous studies have demonstrated the occurrence of AF in congestive heart failure and in myocardial infarction [26]. We agree with Movahed *et al.* [22] that it is possible that coronary microvascular disease with ischemia (inducing metabolic stress) could be involved in the development of AF.

### AF and CAD

In this study, the overall incidence of patients with CAD was 34%. Lip and Beevers [10] described an incidence of 46% and stated that CAD is one of the commonest causes of AF in the western world. Watson *et al.* investigated 121 outpatients with pacemakers capable of arrhythmia detection and found an incidence of CAD of 40% in the patient group with >50% time in AF [27]. On the other hand, Van Gelder *et al.* (investigating only patients with permanent AF) detected an incidence of CAD of 18% [18]. Crijns *et al.* [8] found an incidence of 24% and another study focusing on middle-aged (50±9 years) asymptomatic subjects with AF, reported an incidence of 22% [19]. All those studies focus on different patient groups, whereas in this study, symptomatic patients with angina were also included, as well as all types of AF. We found an overall incidence of CAD of 34%, which is in accordance with the incidence of 38% reported by the AFFIRM study [9] and the incidence of 30% reported by the ATHENA trial investigating dronedarone [12] - although this trial included only patients with paroxysmal or persistent AF. The incidence of CAD in patients with AF (36.3%) described by Krahn *et al.* [11] is also in accordance with our data. Interestingly, Krahn *et al.* found the strongest relative risk for AF at the onset of ischemic heart disease, diminishing over time [11]. For a better general survey, reported incidences are depicted in Figure 1.

The Framingham study reported that CAD was noted in 25% of the men who later had AF, but prevalence in the controls was 15%, so data did not reach statistical significance [3]. Importantly, a few months later additional Framingham data showed that the combination of AF and CAD adversely affected the prognosis regarding total mortality and that men with CAD had a statistically significant doubled risk of developing chronic AF [7]. Regarding gender, in this study there was a higher incidence of men among the patients with CAD, but data did not reach statistical significance (72% *vs* 60%, p = 0.057, Table 1). Only in the comparison of patients with PCI *versus* patients without CAD or stable CAD, male gender was significantly more common in the PCI group (77% *vs* 60%, p = 0.028, Table 3). However, CAD is commonly associated with AF and maybe this association is somewhat stronger in males.

### AF and need for revascularization

In addition to previous data, our study differentiates between patients with stable CAD (13%) and patients undergoing PCI (21%). Previously, AF had already been related to the severity of infarction [28], [29] and described as an independent predictor of death in patients with left main CAD undergoing primary PCI [30]. The GISSI-3 study [4] analysed 17,944 patients after acute myocardial infarction and determined an incidence of in-hospital AF of 7.8%. Wilhelmsen *et al.* [6] diagnosed myocardial infarction in 34.2% of their male patient cohort before or after the diagnosis of AF. Chest pain during exertion was diagnosed in another 11.8% of men at baseline, comparable with the incidence of stable CAD (13%) in our study. Considering these incidences and the data presented in this study, especially before performing ablation therapy of AF in patients with palpitations and/or chest pain, CAD as underlying reason for these symptoms should be considered.

### AF and location of stenosis

It is often speculated that atrial ischemia plays an important pathophysiological role in the genesis of AF. Hence significant stenosis in the proximal right coronary artery and the circumflex artery prior to the take-off of the atrial branches should increase the likelihood of AF in these patients. Previous studies report that only 43% of patients with CAD and AF showed a diseased right artery and/or circumflex branch of the left coronary artery [31]. Further, in only 65% of these patients was coronary stenosis localized before the take-off of atrial branches [31]. Comparing these results with our data, in patients with stable CAD no significant difference in the allocation of coronary artery stenosis among LAD, LCX and RCA was found. Interestingly, in patients undergoing PCI, CAD was more frequently detected in the RCA. AF may present as a consequence of its symptoms [32], but on the basis of this data no answer can be given whether revascularization of the RCA might contribute to less frequent episodes of AF. The precise group of patients in which CAD might be causal for AF remains unclear and further investigations are needed.

### AF and treatment with a class Ic antiarrhythmic drug

The data of the present study also have some implications for the selection of patients for treatment with flecainide and propafenone. The current AHA/ACC guidelines for the management of patients with AF recommend flecainide and propafenone for a single oral bolus dose (class IIa, “pill-in-the-pocket” concept), stating that these drugs should not be used in patients with CAD [20]. The incidence of “lone AF” in patients with persistent AF is 20–25% [33] and CAD should be taken into account as an underlying heart disease important for the consideration of ablation therapy ([20] Fig. 12) and the choice of the antiarrhythmic drug ([20] Fig. 11). At least a stress test is reasonable in patients with signs or risk factors for CAD before administering an antiarrhythmic agent [20], considering that recurrent or chronic ischemia can also lead to impaired left ventricular conduction and thereby cause proarrhythmic effects of class IC antiarrhythmic drugs. Our data underline this recommendation and importantly provide data regarding the incidence of patients with AF undergoing PCI (21%) – a patient group which was identified as “high-thromboembolic-risk”, even having a CHADS_2_≤1 [34].

The results of the present study may give rise to further investigations, which are needed to elucidate the exact incidence and pathophysiological role of CAD in patients with AF.

### Limitations

First, the non-randomized design of this single centre investigation might have potentially influenced the comparative analysis. Second, similar to previous studies, the results of this study are limited to hospitalized subjects. While caution is therefore needed in the interpretation of our data, we consider it improbable that these limitations have influenced our main findings.

### Conclusions

In patients with AF presenting without previously diagnosed or excluded CAD, the overall incidence of CAD was relatively high at 34%. The incidence of patients undergoing PCI was 21% and the incidence of CAD in patients >70 years was even 41%. Increasing incidence of CRF in the western world should lead to a careful investigation respecting the FRS in patients presenting with AF to either definitely exclude or establish an early diagnosis of CAD. This could contribute to an early and safe therapeutic strategy considering the initiation of type Ic antiarrhythmics and oral anticoagulation.
